# Missing in action: the right to the highest attainable standard of mental health care

**DOI:** 10.1186/s13033-022-00537-8

**Published:** 2022-06-11

**Authors:** Yun Ju C. Song, Sebastian Rosenberg, Belinda Smith, Jo-An Occhipinti, John Mendoza, Louise Freebairn, Adam Skinner, Ian B. Hickie

**Affiliations:** 1 The Brain and Mind Centre, Faculty of Medicine & Health, University of Sydney, Building F, Level 54, 94 Mallet Street, Camperdown, NSW Australia; 2 Computer Simulation & Advanced Research Technologies (CSART), Sydney, Australia; 3 Sydney Law School, University of Sydney, Camperdown, Australia; 4 Health and Sport Science, University of Sunshine Coast, Sippy Downs, Australia; 5 Research School of Population Health, Australian National University, Canberra, Australia; 6 Mental Wealth Initiative, University of Sydney, Camperdown, Australia

**Keywords:** Mental health, Human rights, Discrimination, Mental health services, Access, International obligations, Legislation, Mental health standards, Disability

## Abstract

**Background:**

The right to the highest attainable standard of mental health remains a distant goal worldwide. The Report of the UN Special Rapporteur on the right of all people to enjoyment of the highest attainable standard of physical and mental health pleaded the urgent need for governments to act through appropriate laws and policies. We argue that Australia is in breach of international obligations, with inadequate access to mental health services, inconsistent mental health legislation across jurisdictions and ongoing structural (systematic) and individual discrimination.

**Discussion:**

Inadequate access to mental health services is a worldwide phenomenon. Australia has committed to international law obligations under the Convention on the Rights of Persons with Disabilities (CRPD) to ‘promote, protect and ensure the full and equal enjoyment of all human rights and fundamental freedoms by all persons with disability, with respect to their inherent dignity’. This includes people with mental health impairment and this convention includes the right to ‘the highest attainable standard of mental health’. Under the Australian Constitution, ratification of this convention enables the national government to pass laws to implement the convention obligations, and such national laws would prevail over any inconsistent state (or territory) laws governing mental health service provision.

**Summary:**

The authors argue that enabling positive rights through legislation and legally binding mental health service standards may facilitate enhanced accountability and enforcement of such rights. These steps may support critical key stakeholders to improve the standards of mental health service provision supported by the implementation of international obligations, thereby accelerating mental health system reform. Improved legislation would encourage better governance and the evolution of better services, making mental health care more accessible, without structural or individual discrimination, enabling all people to enjoy the highest attainable standard of health.

## Background

### The international obligations of the right to health

In ratifying international conventions on human rights, a nation publicly and formally agrees to recognise and fulfil those rights. In respect of mental health, Australia agreed to recognise ‘the right of everyone to the enjoyment of the highest attainable standard of physical and mental health’ over three decades ago by ratifying the *International Covenant on Economic, Social and Cultural Rights* (ICESCR), which contains this right in Article 12 [1]. The Australian government agreed to ‘take steps… to the maximum of its available resources, with a view to achieving progressively the full realization of the rights …by all appropriate means, including particularly the adoption of legislative measures’ (Art 2). Further, the *International Covenant on Civil and Political Rights *(*ICCPR*) also articulates that there is a free-standing right to non-discrimination, not subject to the maximum of available resources limitation (Art 2 and Art 26) [2]. This right was given significantly more meaning and force by the *Convention on the Rights of Persons with Disabilities* (CRPD), which Australia ratified in 2008. However, the terms of international conventions do not have legal force in Australia unless Parliament incorporates them into legislation [3]. Further, Australia has interpreted the CRPD to still permit ‘compulsory assistance or treatment’ of persons with disabilities and that it does not cover non-nationals seeking to enter or remain in Australia.

The CRPD has four notable attributes. Firstly, it has a particular degree of legitimacy, having been created with the greatest civil participation of all the human rights conventions, and specifically, involvement of over 800 representatives of disability organisations [4, 5]. Related to this (and secondly), the CRPD represents a fundamental shift from seeing persons with disability as merely objects of rights, entitled only to be protected and cared for, toward subjects of all human rights [6]. Thirdly, the CRPD promotes a social model of disability, which conceptualises disability not as a deficit, but as social or disabling barriers created by structures and practices in society and the right to have these removed [5, 7]. Finally, the CRPD sets out fundamental guiding principles for governments in fulfilling human rights (Art 3). The CRPD requires ratifying countries to promote and protect the rights of persons with disabilities and ensure equality of treatment and outcomes [8].

Specifically, Article 25 of the CRPD recognises that persons with disabilities have the right to the enjoyment of the highest attainable standard of health, without discrimination based on disability [9]. The question for each country then is not whether someone with a mental health disability has a right to the highest attainable mental health, but what governments need to do to ensure that this right is fulfilled. This international obligation requires governments not only to provide access to health services to those with disability, but to ensure those services are provided in ways that acknowledge disability and are adapted to provide equal protection and benefit.

Globally mental illness is the largest single cause of disability, accounting for 14.6% of years lived with disability [10]. Mental and substance use disorders were the leading contributor to years lived with disability than any other category of disease in Australia [11]. More than 15 years have passed since ‘*Not for Service*’ was jointly released by the Human Rights and Equal Opportunity Commission (now Australian Human Rights Commission), the Mental Health Council of Australia (now Mental Health Australia), and the University of Sydney. A succession of seminal reports and inquiries have found that Australians with mental illness struggle on a daily basis to access appropriate health care or be treated with dignity when they enter the mental health system [12, 13]. More recently, the COVID-19 pandemic has highlighted stark inequities in our economic and political systems and the devastating impact of systemic discrimination [14]. The undermining of human rights remains, when mental health care services are based on discriminatory laws and practices [15].

It is thus unsurprising that a recent analysis of Australia’s performance under the CRPD concluded that Australia has not made adequate progress towards achieving rights for people with disability, with Australia’s interpretive declarations preventing reform and permitting violations of international obligations [16]. Compliance with the CRPD is complicated by Australia’s federated system of governance; it is the state and territory governments, not the federal (national) government, that are responsible for delivering many of the services that give effect to Australia’s obligations under the CRPD. Australia’s federal, state, territory and local governments in fact share responsibility for mental health service provision [17]. Multiple reports, inquiries and reviews suggest this fragmentation and role confusion to be one of the reasons Australia’s mental health system is in crisis [12, 13].

Individuals that experience oppression and discrimination may feel empowered to be supported by international bodies, rather than their own national governments [18]. An example of this may be reflected by the functions of the Committee of the CRPD, which is the body of independent experts that monitors the implementation of the CRPD. Australia has also signed the CRPD’s Optional Protocol in 2009, which allows individuals to directly complain to the Committee [19]. Australia reported to this Committee in 2013 and 2019. Both reports highlighted significant levels of inequality, discrimination and segregation experienced by people with disability [20, 21]. Little changed in the 6 years between each report, showing that people with disability continued to experience poverty, disadvantage and human rights violations [22]. The Committee’s role is to monitor and keep nations accountable. It requested Australia withdraw its interpretive declarations and repeal laws, policies, and practices in breach of the CRPD. This has not yet occurred [16, 23]. When their directives are ignored by countries, the role of such Committees are undermined [18]. These Committees can support individuals by allowing them to access the international human rights law system in a meaningful way [24, 25]. However, the main challenge is enforcement of Committee decisions by States, given these Committees are not judicial organs and can only make recommendations to which States can choose to implement or not [26].

An international obligation on a government not to discriminate might not translate into anti-discrimination laws, which tend to focus on prohibiting citizens from discriminating against each other, rather than the positive rights as expressed in the CRPD. Such anti-discrimination measures might also be pursued under the ICESCR and ICCPR [1, 2]. In Australia, complaints about disability discrimination constitute the highest number of complaints made to the Australian Human Rights Commission (AHRC) [22]. Justiciability can provide another mechanism to ensure the fulfilment of international commitments, however, such commitments do not always translate into rights that can be claimed and adjudicated upon, leaving them merely as policy objectives [27]. There have been limited challenges to legal decisions involving the allocation of mental health resources and unsuccessful attempts at law reform, however, the recognition of international human rights standards may enable a more effective mechanism for achieving change [27].

### Using the law to fulfil international obligations

#### Discrimination law

Ratification of the CRPD committed Australia to use ‘all appropriate means, including the adoption of legislative measures’ to fulfil its obligations. The key legislative measure Australia has cited as evidence of implementation of CRPD obligations is the *Disability Discrimination Act 1992* (Cth) (DDA). This statute was enacted before ratification of the CRPD but amended in 2009 to make clear that the Act was aiming to ‘give effect to the CRPD’, reflecting some of the obligations of the CRPD. These amendments to the DDA importantly included the recognition of the CRPD, including explicit references to the CRPD, aiming to ‘give effect to the CRPD.’ Similar statutes to the DDA exist in each state and territory. These laws prohibit disability discrimination in various fields, such as employment, education and the provisions of goods and services, including health services [28]. This legislation is important and can be used to challenge discrimination on the grounds of disability. While the DDA is an important tool for implementing the CRPD, in respect to Article 25, as described below, it is clearly not adequate.

#### Individual enforcement

Anti-discrimination laws in Australia, including the DDA, primarily afford individual civil rights; they are not enforced by a public agency. The *Australian Human Rights Commission Act* 1986 (Cth) governs the process for making complaints [29]. Enforcement of the law is entirely dependent upon individual victims of discrimination bringing complaints; no public agency is empowered to identify and publicly prosecute breaches [30]. The first step is conciliation at the AHRC. If the matter is not settled by conciliation, the complainant can proceed to litigate in a federal court [28], but this poses barriers such as formality, evidentiary burden on complainants, legal costs and, if the case is unsuccessful, an order to pay the other side’s legal fees as well [29]. Advocacy groups argue that complaints about health services may trigger victimisation of the complainant and compromise the ongoing care they receive or their future access to these services. Most individuals would be inhibited from raising a complaint, including a discrimination complaint, due to concerns about self-disclosure, stigma and fears that the stress of the process itself may trigger illness relapse [31].

All complaints made to the Commission must be referred to the President of the Commission [28], but the legislation is silent on when this must occur and how long the process should last. Once the complaint is lodged with the President it could take up to 12 months for resolution, arguably an intolerable period of uncertainty for anyone with a disability. The lack of agency enforcement or support, and a complaints process that is lengthy and costly to the complainant may help explain why discrimination persists [32]. It is ironic that a law designed to address disadvantage puts the burden for enforcement on those disadvantaged individuals. The focus on individual claims also means systemic problems with services are less likely to be challenged. Noting that equality under the CRPD calls for special measures or supports to enable rights to be realised, government support for pressing claims should be considered.

The Victorian Mental Health Complaints Commissioner illustrates how an agency might operate as an advocate to support complainants to investigate and resolve complaints [33]. Seven of Australia’s nine jurisdictions have opted for a mental health commission, however, Victoria’s mandate is unique, providing an accessible and approachable process whereby complaints are to be resolved, safeguarding rights for the complainant, improving services and individual experiences to prevent a recurrence of issues [34]. The Commissioner can also obtain responses from services, investigate issues and make recommendations regarding quality and safety issues identified [35]. Such agencies could be given additional powers and resources to support people with mental illness to identify and press discrimination complaints.

#### Enactment of disability health standards

The DDA prohibits discrimination but does little to help mental health service providers avoid discrimination and adopt a human rights approach. However, the DDA does provide a mechanism for elaboration and certainty, in the form of ‘disability standards’ [28]. There are already such disability standards in relation to public transport, education and access to premises [36–38]. The introduction of transport disability standards led to significant improvements in accessibility [31, 39]. However there are no legislative standards that apply to other services. The provision of goods and services has generated the second highest number of DDA complaints after employment, many from people with mental illness [40].

It is important to appreciate that the DDA standards are legally enforceable statutory instruments, however not all standards have this force and effect. Agencies such as the Australian Commission on Safety and Quality in Health Care have adopted National Safety and Quality Health Service Standards (NSQHSS) to develop an agreed understanding of what is expected or required for quality health care [41]. Consumer rights were a key feature of the very first national mental health strategy in 1992 [42], and the National Standards for Mental Health Services (NSMHS) were established in 1996 to promote these rights by encouraging providers to understand key components of mental health service [43]. However, the mapping of the NSQHSS against the NSMHS found discrimination through the inequity of access to mental health services [44]. State and territory mental health services annually report their success in meeting national quality standards for service, yet widespread evidence of poor care or breaches of human rights persist [45]. Further, the implementation of NSMHS in public and private health services remains a discretionary decision of state and territory health departments [41, 43]. Although first proposed as a major milestone under the Mental Health Strategy in 1997, the standards are not mandatory and fail to demonstrate their capacity to drive systemic quality improvement or accountability.

The NSQHSS could inform and underpin the development of DDA standards for (mental) health services. The DDA standards are legally enforceable, could prevail over inconsistent state and territory legislation, and are designed to promote substantive equality. The DDA mental health standards could be used as a tool to drive greater consistency, transparency, and the quality of each jurisdiction’s practices. This kind of reform, where the monitoring and enforcement of disability standards is incorporated into existing regulatory processes has already been recommended [31]. The formulation and enforcement of disability standards amended under the DDA would render breaches unlawful discrimination. However, the development and implementation of standards is not always simple. State and territory requirements differ, including alignment with their respective Mental Health Acts. An alternative approach would be for the states and territories to work cooperatively to adopt the same standards in their own jurisdictions. Inconsistencies in enforcement and interpretation would need to be carefully considered. This kind of approach would no doubt incur costs, but the benefits could far outweigh the costs associated with applying or complying with the DDA [31]. Mental Health Service Standards under the DDA could incorporate a requirement to periodically review the efficiency and effectiveness of the standards, as evidenced in the transport standards. Legislatively binding the review and effectiveness of such standards may provide duty bearers with consistent national guidelines and self-regulatory benchmarking. Public reporting may also ensure a process of systemic quality improvement and accountability, encouraging the highest attainable health care to be accessible to those who need it most.

#### Consistency in the law

A factor identified as undermining Australia’s outcomes in mental health is legislative inconsistency across states and territories [40, 46]. For example, each jurisdiction’s mental health laws are generally silent on access to services. However, in the Northern Territory there is separate legislation which allows their Mental Health Tribunal to review decisions on the refusal of access to a mental health facility [47]. Legislating consistent rights across jurisdictions may strengthen the rights of those who struggle to access mental health services in an already inconsistent and fragmented mental health system [8]. In line with their ‘positive duty’, Governments must do more than just prohibit discrimination by services providers. They must review and revise the laws under which these services are provided, to ensure consistency and accountability. For example, the recent Royal Commission into Victoria’s Mental Health System highlighted human rights as fundamental to achieving its aims, reporting that current arrangements in that state do not represent the ‘gold standard’ in promoting and protecting human rights [48].

The fragmented nature of Australian mental health care is borne out in its legislation. Each state and territory has its own *Mental Health Act* [33, 35, 47, 49–53], and they are inconsistent in significant ways. There are legislative barriers for example, that make it difficult to transfer patients under compulsory treatment orders from one state to another. Some states make explicit reference to a complaints process regarding service provision (Table 1), others do not. For example, *The Mental Health Act 2007* (NSW) [52] is silent on a complaints process, whereas in Western Australia [35] there are specific provisions regarding processes for complaints about mental health services. In South Australia there is separate legislation covering complaints against health or community service providers [54]. Whether people with mental illness understand the legislation they have recourse to, and this varies depending on where they live in Australia. Drawing on the framework in Victoria, a consistent national complaints process across jurisdictions in partnership with all Mental Health Commissions may provide a non-threatening, accessible process.

**Table 1:** Inconsistent complaints process across jurisdictions

Mental Health Acts in Australia	Complaints process
Mental Health Act 2014 (WA)	A complaint is to be brought directly to the mental health service provider or a complaint to the Director under the Health and Disability Services (Complaints) Act 1995 (WA)
Mental Health Act 2014 (VIC)	The Mental Health Complaints Commissioner (MHCC) was created under the Mental Health Act 2014 (the Act) to help safeguard and promote the rights of consumers, carers, families, and support people in Victoria. The Act includes 12 mental health principles that must be upheld by staff of public mental health services
Mental Health Act 2009 (SA)	No specific complaints process for mental health service provision, rather complaints are governed through the Health and Community Services Complaints Act 2004
Mental Health Act 2016 (Qld)	No legislative direction on the process of complaints but only a directive to the Office of the Chief Psychiatrist can investigate serious matters concerning the administration of the Mental Health Act including the rights of patients
Mental Health Act 2007 (NSW)	No complaints process referenced
Mental Health Act 2015 (ACT)	No complaints process referenced
Mental Health Act 2013 (TAS)	No complaints process referenced
Mental Health and Related Services Act 1998 (NT)	Internal complaints procedure for mental health service provision

Therefore, rather than reform focusing on litigation for enforcement, standard setting has been suggested as an alternative to improve compliance with consistent standards [30]. Organisations and service providers that are subject to standards are likely to experience greater certainty about how to comply, potentially leading to reductions in litigation, compensation, and other costs [31].

## Conclusion

Human rights conventions may not end inequity but they have provided benchmarks by which to measure the words and practices of governments and other actors [55]. If international obligations are to have significance, they must fundamentally guide incorporation of human rights protection into domestic legislation (and practice). For example, the DDA could include a positive duty to enable equality, not merely a negative duty to not discriminate, particularly on the provision of mental health services and the inclusion of mental health standards [56]. For international law to have relevance, countries must take their obligations under international conventions seriously.

The authority of international obligations can pave the way for genuine mental health reform, a process that has thus far proved difficult. The CRPD was intended to be a shining light of social transformation, aimed at removing barriers that exclude people with disabilities from the benefits of citizenship [16, 57]. Anti-discrimination laws such as the DDA are purported to provide Australians with a disability the right to substantive equality in critical areas such as employment, education and the provision of goods and services [31]. The affordability and accessibility of health services are particularly vital for those with mental ill-health [58]. However, despite decades of attempts to pursue successful mental health system reform, any person seeking mental health care still runs the serious risk that their basic needs will be ignored, trivialised, or neglected [12]. Legislative changes to reflect the positive obligations of Article 25 would support individuals to access the highest attainable standard of health, in the least restrictive environment [40].

Thirty years after Australia’s first National Mental Health Strategy, the unwillingness to legislate for substantive equality for those with a mental illness is a significant barrier to meaningful reform [58]. Australia is an example of how its legislation is inadequate in its prohibition, with its ongoing failure to legislatively address the critical provision of mental health services for the single largest disability group. Recent inquiries highlight the crisis and the systemic failure of the mental health system [59]. The most recent Report of the Special Rapporteur called for a mandate to address the intersectional nature of discrimination, to move away from laws and policies that still use “single-axis” models of discrimination, and fail to properly reflect the lived experience of discrimination [60]. This kind of reform would provide a framework of substantive equality and a rights based approach, requiring governments to be legislatively aligned with relevant international obligations [61–63]. This would equip mental health service providers to deliver the highest attainable standard of health, which in turn would be a step toward promoting ‘mental health and the full enjoyment of all human rights’, satisfying the right of mental health consumers to enjoy full participation in society without discrimination (64).

## Data Availability

Not applicable.

## Abbreviations

CRPD: Convention on the Rights of Persons with Disabilities
AHRC: Australian Human Rights Commission
DDA: *Disability Discrimination Act 1992* (Cth)
NSMHS: National Standards for Mental Health Services
NSQHSS: National Safety and Quality Health Service Standards
MHCC: Mental Health Complaints Commissioner
